# The Consequences of Precipitation Seasonality for Mediterranean-Ecosystem Vegetation of South Africa

**DOI:** 10.1371/journal.pone.0144512

**Published:** 2015-12-09

**Authors:** Michael D. Cramer, M. Timm Hoffman

**Affiliations:** Department of Biological Sciences, University of Cape Town, Cape Town, South Africa; University of California Davis, UNITED STATES

## Abstract

Globally, mediterranean-climate ecosystem vegetation has converged on an evergreen, sclerophyllous and shrubby growth form. The particular aspects of mediterranean-climate regions that contribute to this convergence include summer droughts and relatively nutrient-poor soils. We hypothesised that winter-precipitation implies stressful summer droughts and leaches soils due to greater water availability (i.e. balance between precipitation and potential evapotranspiration; P–PET) during cold periods. We conducted a comparative analysis of normalised difference vegetation indices (NDVI) and edaphic and climate properties across the biomes of South Africa. NDVI was strongly correlated with both precipitation and P–PET (r^2^ = 0.8). There was no evidence, however, that winter-precipitation reduces NDVI in comparison to similar amounts of summer-precipitation. Base saturation (BS), a measure of soil leaching was, however, negatively related to P–PET (r^2^ = 0.64). This led to an interaction between P–PET and BS in determining NDVI, indicating the existence of a trade-off between water availability and soil nutrients that enables NDVI to increase with precipitation, despite negative consequences for soil nutrient availability. The mechanism of this trade-off is suggested to be that water increases nutrient accessibility. This implies that along with nutrient-depauperate geologies and long periods of time since glaciation, the winter-precipitation may have contributed to the highly leached status of the soils. Since many of the ecophysiological characteristics of mediterranean-ecosystem flora are associated with low nutrient availabilities (e.g. evergreen foliage, sclerophylly, cluster roots), we conclude that mediterranean-climates promote convergence of growth-forms in these regions through high leaching capacity.

## Introduction

Despite their small spatial extent (5% global land surface [1]), mediterranean-climate ecosystems including maquis (Mediterranean Basin), chaparral (California), matorral (Chile), fynbos (South Africa) and mallee and kwongan (Australia) are home to 20% of global vascular plant species, and are associated with large numbers of rare and endemic plants [2]. Although the flora of these mediterranean-climate regions are largely unrelated, these ecosystems exhibit a high degree of convergence on growth-forms that are evergreen and sclerophyllous trees and shrubs [3, 4, 5], but more commonly shrubs, especially at lower precipitation [6]. Part of the Greater Cape Floristic Region (GCFR) occurs in the mediterranean-climate region of southern Africa [7] and comprises fynbos, strandveld and renosterveld (all subsumed in the Fynbos biome, henceforth Fynbos), the Succulent Karoo and Afromontane forests [8]. These diverse vegetation types are associated with a wide range of geologies, topographic variability and strong climatic gradients, which all contribute to niche heterogeneity and potentially to the maintenance of the high species richness in the GCFR [9], which has accumulated over long time periods [10].

Although definitions vary [3, 11], mediterranean-climates are generally defined as having an average temperature of the coldest month less than 18°C, and 8 to 12 months with average temperatures exceeding 10°C [1]. In addition, 3-fold more rain falls in the winter half-year than during the summer half-year, precipitation in the driest month is less than 30 mm and mean annual precipitation (MAP) is less than 890 mm. A strongly mediterranean-climate may thus impose severe water deficits in summer when temperatures are appropriate for growth. In contrast, water is abundant in winter when temperatures and light availability are low, resulting in partial asynchrony between resource availability and growth [12]. These circumstances are thought to have resulted in a number of evolutionary specializations of the GCFR flora. These include evergreen and sclerophyllous leaves that may confer a degree of drought tolerance [13], coupled with small leaves that promote water loss in winter and consequently nutrient transport through soil to roots by mass-flow as well as heat-loss without necessarily concomitant water-loss in summer [14]. Succulence is also a common specialization, especially in more arid areas [15]. While shallow-rooted species are forced to be anisohydric and constrain growth to favourable hydrological periods, deep-rooted isohydric species are able to access water well into the dry season [16, 17], enabling partial escape from constraints of precipitation seasonality. Growing season normalised difference vegetation index (NDVI) maxima for the Fynbos occur in both spring (Aug) and summer (Jan) [18], possibly as a consequence of both the diverse strategies to cope with the mediterranean-climate and the fact that this biome extends into areas where precipitation is less seasonal, and even dominated by summer-precipitation in the east [19].

Mediterranean-ecosystem soils are generally nutrient poor, although the particular nutrients that are poorly available vary within the winter-precipitation region [20, 21]. Soils are the combined product of regional climate, biota, topographic relief, parent geology, soil age [22] and deposition. Soil development generally follows a predictable pattern described by ‘Albrecht’s curve’ in which base saturation (BS; the sum of base cations divided by cation exchange capacity), clay and cation exchange capacity (CEC) initially increases with weathering and then decreases, particularly when precipitation exceeds evapotranspiration [23, 24]. In contrast, P decreases with time since the onset of pedogenesis [25, 26, 27]. The soils of the GCFR are heterogeneous due to a combination of complex geomorphic processes and variable underlying geologies [9] together with potential transformations generated by vegetation (e.g. the putative role of plants in the formation of laterites in south western Australia [28]) and deposition [29, 30]. The long periods of stable environmental and geological conditions have subjected the soils to a long history of leaching, resulting in what have been referred to as old, climatically-buffered, infertile landscapes (OCBILs) [31].

Although the soils of the GCFR are highly heterogeneous, some component soils are especially nutrient-depauperate. These include highly weathered sandstone-derived soils that have been leached of cations, and in which total P is especially low [32]. Many GCFR soils also have low total N concentrations [32], and are extremely heterogeneous with respect to total N [33]. Although the highly leached soils of the GCFR are low in most nutrients, P is likely to be the most limiting nutrient (*sensu* Vitousek et al. [34]; i.e. P addition transforms ecosystems). Across diverse plant families, GCFR species show higher foliar N:P ratios than do plants from the world’s other winter-precipitation ecosystems [32]. The vegetation of the GCFR is, however, adapted to these conditions [35] and may even suffer toxicity with elevated supply of nutrients [36]. Adaptations to low nutrient status of these soils include the occurrence of N_2_–fixing legumes, parasitic and carnivorous plants, plants with cluster roots and functionally analogous structures, diverse mycorrhizae for P uptake, small leaves that promote water loss in cold wet conditions, sclerophyllous leaves, serotiny and resprouting growth habits that enable nutrient conservation (among others traits, [21]). Together these adaptations are strongly associated with the evergreen and shrubby growth-habit of the floras.

Although water and nutrient concentrations are commonly considered as separate resources, they do interact in determining the availability of nutrients [37], and consequently in controlling species occurrences and plant biomass. This interaction has complex mechanistic underpinnings, because both water and nutrients are important constraints for plant growth. For example, water may promote biomass accumulation in forests with consequences for soil development, such as accumulation of organic carbon and increased microbial flora, leading to edaphic development favouring particular flora in what has been termed “niche construction” [38]. Nutrient mobility in soils is also determined by water availability because it is the medium in which nutrients diffuse and because transpirational consumption of water powers mass-flow of nutrients towards roots and from roots to shoots [37]. Thus the role of water in nutrient mobility provides a mechanism for water availability to influence nutrient accessibility. For example there was an interaction between water availability and soil total P concentrations in determining maximum tree height in south-west Australia [39], in which tree height on low-P concentration soils could be high if water availability was high, and conversely, if water was scarce but P concentration was high. As a consequence a trade-off exists between water and nutrient availability in which water and nutrient availability are partially inter-changeable.

Here we focussed on the consequences of a mediterranean-climate for NDVI as a measure of green biomass [40] of biomes in the GCFR (comprising Fynbos and Succulent Karoo biomes) in comparison to other biomes of South Africa. Globally [41] and in South Africa [42] NDVI is strongly positively correlated with MAP. Plant traits in mediterranean-climate ecosystems have also been suggested to be more strongly associated with high precipitation-reliability than with nutrient impoverished soils [5]. Globally, however, net primary productivity (NPP) tracks the unimodal pattern of soil fertility with increasing water availability [24]. We thus hypothesised that mediterranean-climate winter-precipitation that exceeds evaporative demand may contribute to leaching of soils and decreased nutrient availability, resulting in an interaction between the direct positive effects of precipitation and the indirect negative influences of precipitation operating through nutrient availability on leafy biomass. While the role of leaching in pedogenesis is well known [22, 23, 24], the link between mediterranean-climates and leaching has not previously been invoked as an explanation for nutrient impoverishment of these ecosystems. We also attempted to discern the consequences for NDVI of winter- versus summer-dominated precipitation. A correlative approach was followed using NDVI together with vegetation properties, climate and edaphic data for conservation areas of South Africa.

## Methods

### Spatial sampling

**Fig 1:**
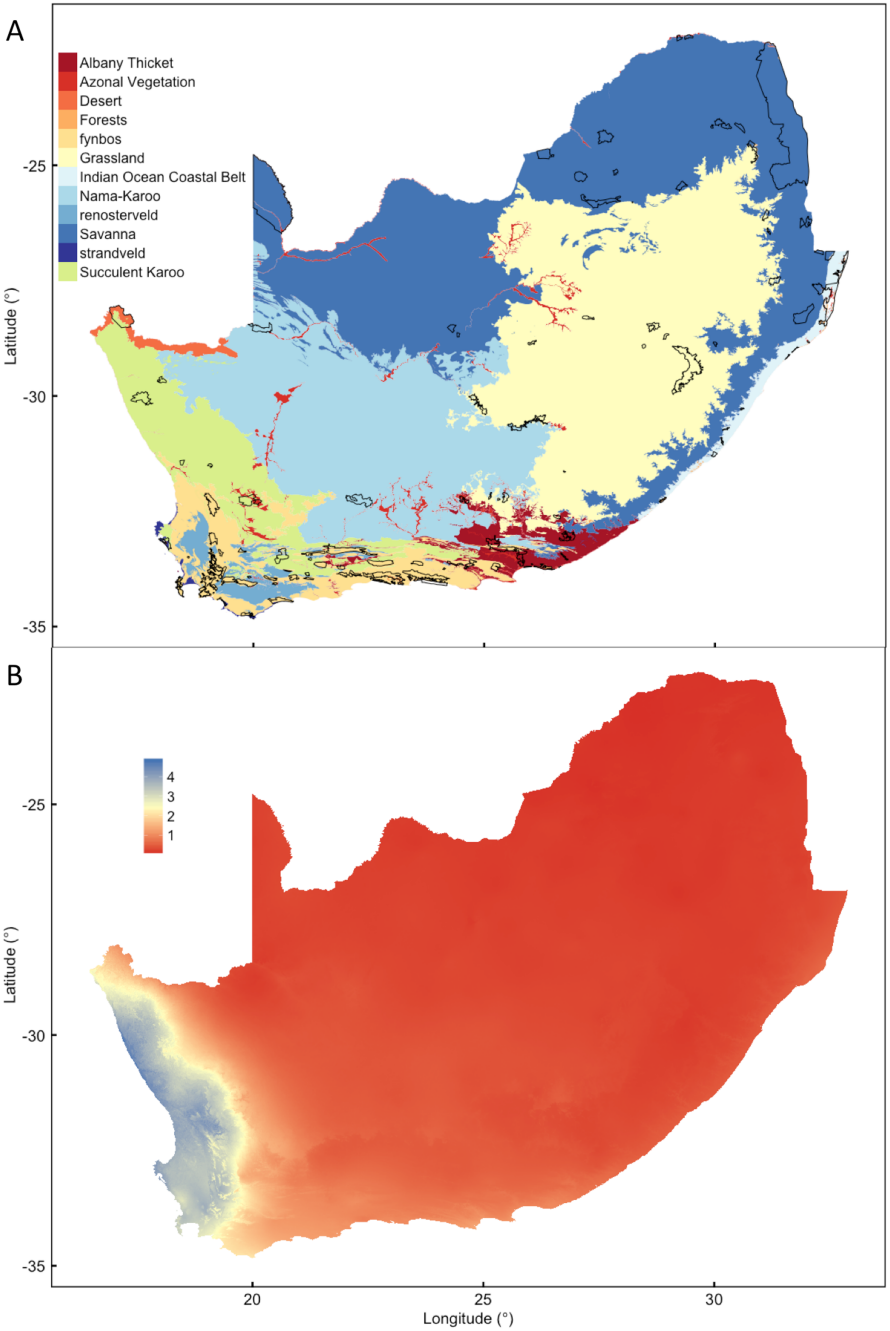
Maps of (A) the biomes of South Africa with the Fynbos biome separated into fynbos, renosterveld and strandveld, and (B) the ratio of winter half-year (Apr–Sep) to summer half-year precipitation. The conservation areas that were sampled using a 1 km^2^ grid of points are indicated.

To avoid sampling transformed or heavily disturbed areas, data for vegetation, climate and edaphic properties were only taken from within conservation areas identified by Mucina and Rutherford [8] as “major parks” within South Africa (Fig 1). Geographical data extraction was conducted using QGIS version 2.2.0-Valmiera [43]. The spatial extents of the conservation areas were overlaid by a 1 km^2^ grid of 53 904 points at which all sampling was then conducted. No attempt was made to average or interpolate data from around each grid point.

### Vegetation, edaphic and geological characteristics

The biomes of South Africa are defined on the basis of the floristic extents of the lower-order “vegetation units” that are included in a particular biome [8]. The spatial distribution of vegetation was obtained by extracting biomes (Fig 1A) identified by these authors for each grid point, but with the Fynbos additionally separated into strandveld, renosterveld and fynbos. This was done because it has been argued that these three vegetations are floristically unrelated [44] and because these three vegetations are climatically and edaphically distinct and important in this analysis. As a consequence of this we have referred to the collection of biomes (names capitalised) and strandveld, renosterveld and fynbos (names lowercase) as “vegetation types”, to differentiate these from the formally defined “vegetation units” designated by Mucina and Rutherford [8].

The Harmonized World Soil Database (HWSD, Version 1.2, [45]) was produced by harmonizing regional soil databases and the FAO-UNESCO Soil Map of the World [46] using a standardized structure imposed on diverse sources of information. HWSD data are available as polygons for soil mapping units, with each polygon associated with textural and chemical characteristics. Each polygon has one or more component soils (‘sequences’). For each “soil mapping unit” (MU) the weighted average of the component ‘sequences’ (SEQ) was calculated based on the proportion (SHARE) of the MU occupied by that sequence (codes refer to variable names used in the database). After finding similar relationships between NDVI and variables for topsoil and subsoil (see below) we elected to use the depth-weighted average of topsoil and subsoil per soil mapping unit value for each soil variable. The variables selected for analysis included pH (water extract), clay fraction (%, w/w), organic carbon (OC, %, w/w), soil cation exchange capacity (CEC, cmol kg^−1^), base saturation (BS, %) and total exchangeable base (TEB, cmol kg^−1^).

The 1:1 000 000 geological map of South Africa was obtained from the Council for Geoscience (Private Bag X112, Pretoria). From these data the stratigraphic description was used as a categorical variable.

### Climate data

Climate data were obtained from Hijmans et al. [47] for the period 1950–2000. These data were generated through interpolation of average monthly climate data from weather stations on a 30 arc-second resolution grid (*ca*. 1 km^2^ resolution). Variables included are elevation, mean annual precipitation (MAP), and mean annual temperature (MAT), mean temperatures (MT) in year-quarters (q) and the precipitation seasonality expressed as the ratio of winter (Apr-Sep) to summer rainfall (Fig 1B). Since the precipitation concentration has previously been associated with African woody cover [48], the Precipitation Concentration Index (PCI) was obtained from Schulze [49]. PCI values of 100% indicate that all the precipitation occurs in one month while a value of 0% indicates equal rainfall throughout the year. Potential evaporation (PET) was obtained from Trabucco and Zomer [50] in which PET was modelled using the Hargreaves et al. [51] method with data from Hijmans et al. [52] and verified by comparison with separate data sources. From the climatic data, the monthly PET was subtracted from monthly precipitation to obtain an index of water availability (P–PET).

### Normalized difference vegetation index

Normalized difference vegetation index (NDVI) data were obtained for southern Africa from eMODIS TERRA produced by US Geological Survey Earth Resources Observation and Science Center. NDVI, which measures chlorophyll in vegetative cover, is defined as (NIR—VR) / (NIR + VR), where NIR is the near-infrared reflectance and VR is the visible-red reflectance [53]. This vegetation product is calculated from MODIS L1B Terra surface reflectances, corrected for molecular scattering, ozone absorption, and aerosols using MODIS Science Team algorithms. The data available are the product of a time series smoothing [54] for data collected between 2001 and 2010. eMODIS 10-day maximum-value composite NDVI images at 250 m spatial resolution were averaged using the QGIS raster calculator to obtain monthly average NDVI values. These monthly data were then averaged to obtain the annual average NDVI.

### Data analysis and statistics

Conservation areas within South Africa are dominated by large reserves within savannas. To eliminate this sample bias, the data were averaged for each unique ‘vegetation unit’, i.e. as described by Mucina and Rutherford [8], occurring with a particular geological stratigraphic description. As a consequence, some vegetation units were sampled repeatedly where these were associated with a number of geological formations. This resulted in 1 116 unique combinations of vegetation and stratigraphy occurring within conservation areas. These vegetation unit-stratigraphic combinations were used directly for analyses across all vegetation types. The climatic and NDVI data were summarized for each vegetation type and subjected to one-way ANOVA.

Predictor variables were screened for colinearity using the “select07” procedure outlined by Dormann et al. [55], and less powerful variables removed where colinearity existed. The retained variables were included in multiple regression analysis of the quadratic predictors of NDVI and BS with stepwise backward-elimination based on the Akaike Information Criteria (AIC). We also examined dependence of NDVI on water availability and BS for each vegetation type individually using multiple regression.

Boosted regression trees (BRT) provide a machine learning-based model of response variables, and do so without involving normal null-hypothesis significance testing. BRT model construction was performed, as detailed by Elith et al. [56] and implemented in R [57]. Models for NDVI were constructed with water availability and the edaphic variables (see above) using the ‘dismo’ package version 0.7–23 (Hijmans et al. 2012). Tree complexity (5) and learning rate (0.01) were optimised for the analysis and a bagging fraction of 0.5 was used. After initial BRT analysis, the model was simplified following procedures outlined by Elith et al. [56]. The BRT analysis was used to rank the importance of different predictor variables in determining the NDVI.

A structural equation model (SEM) was constructed to evaluate the relative strength of the influence of water and soil properties on NDVI. The SEM was produced using backward-simplification based on the AIC score of quadratic multiple regression of NDVI on water-related measures (P-PET, precipitation seasonality and PCI) and soil related measures (BS, TEB, CEC and organic C and clay) to yield two composite variables which were included in the SEM implemented using the package Lavaan [58] in R [57].

## Results

### Vegetation type-climates

The twelve vegetation types of South Africa analysed here occur in a temperate climate with mean annual temperature (MAT) range (5 to 95 percentiles) of 12.4–23.8°C (Table 1), with the consequence that snow and permafrost that complicate global analyses are limited in the region. The fynbos, renosterveld, strandveld and Succulent Karoo vegetation types have distinctly lower temperatures during the wettest quarter, relative to the other vegetation types. These vegetation types occur across a range of mean annual precipitation (MAP; 70–1014 mm), but together with Desert have more than 30% of average precipitation in the coldest quarter. Despite the common perception that fynbos, renosterveld and Succulent Karoo are mediterranean-climate vegetation, the average ratio of winter to summer precipitation of these are lower than the threshold for inclusion in the definition of a mediterranean-climate (i.e. winter: summer precipitation > 3; [1]). Strandveld does, however, fall into this definition of a mediterranean-climate region, as do components of fynbos, renosterveld and Succulent Karoo as indicated by the fact that 95 percentiles of the winter:summer precipitation ratios exceed the threshold (Table 1) and the fact that the ranges of these latter vegetation types extend further east than the strictly mediterranenan-climate region (Fig 1A *cf*. Fig 1B).

**Table 1:** Climate characteristics associated with vegetation types of South Africa.

Vegetation	n	MAT (°C)	MT wettest q (°C)	MAP (mm)	Proportion MAP in coldest quarter (%)	Winter/Summer precipitation
Savanna (S)	339	17.7 (20.8) 23.1 h	21 (25) 27 e	413 (628) 1013 e	2 (5) 11 a	0.14 (0.22) 0.4 a
Nama-Karoo (Nk)	47	15.6 (19.5) 21.2 fg	20 (25) 27 e	115 (184) 411 b	4 (6) 14 a	0.24 (0.33) 0.54 d
Grassland (G)	119	10.8 (14.7) 17.4 a	15 (19) 22 d	445 (842) 1241 f	4 (6) 12 a	0.19 (0.26) 0.44 a
Forests (F)	84	14.6 (19.5) 23.8 g	14 (22) 27 f	472 (833) 1138 f	2 (11) 25 b	0.14 (0.48) 1.02 abc
Indian Ocean Coastal Belt (I)	16	18.6 (20.8) 22 h	21 (24) 25 e	877 (992) 1131 g	10 (12) 14 b	0.4 (0.46) 0.51 b
Albany Thicket (At)	65	15.4 (17.5) 18.9 cd	16 (19) 22 cd	223 (403) 550 c	14 (18) 22 c	0.5 (0.72) 0.89 d
Azonal (A)	79	15.9 (18.9) 22.6 ef	13 (20) 27 c	43 (471) 935 d	2 (20) 47 c	0.16 (1.03) 3.64 c
Desert (D)	31	17.6 (18.3) 19.2 de	17 (20) 22 c	43 (57) 74 a	27 (30) 33 d	1.08 (1.27) 1.47 e
fynbos (Ff)	206	12.4 (15.5) 17.6 b	9 (14) 19 a	302 (606) 1014 e	19 (32) 47 d	0.78 (1.84) 3.56 g
renosterveld (Fr)	32	14.1 (15.9) 17.3 b	11 (14) 18 ab	245 (476) 976 cd	21 (33) 47 d	0.83 (1.83) 3.5 f
Succulent Karoo (Sk)	86	15.7 (17.1) 18.7 c	12 (15) 20 b	70 (187) 378 b	20 (38) 49 e	0.82 (2.49) 4 ab
strandveld (Fs)	12	16.4 (17.8) 19.6 cde	13 (15) 16 ab	291 (461) 762 cd	32 (43) 48 f	1.61 (3.11) 4.11 e

Vegetation types are as defined by Mucina and Rutherford [8], but with the Fynbos biome separated into fynbos, renosterveld and strandveld. Values shown are the 5% percentiles, means (in parentheses), and 95% percentiles of points sampled across national parks of South Africa. The mean annual temperatures (MAT), mean temperatures (MT) in year-quarters (q), mean annual precipitation (MAP), proportion of precipitation in the coldest quarter and ratio of precipitation in the winter half-year (Apr–Sep; Cowling et al. 2005) to summer half-year are given. The number of sampling points within each vegetation type representing the averages of each vegetation unit-stratigraphic combination are shown (n). For each environmental variable different letters indicate significant (P < 0.05) differences between vegetation categories as determined from one-way ANOVA with post-hoc Tukey tests. The values are in order of increasing proportion of MAP in coldest quarter.

### Climatic predictors of NDVI

**Fig 2:**
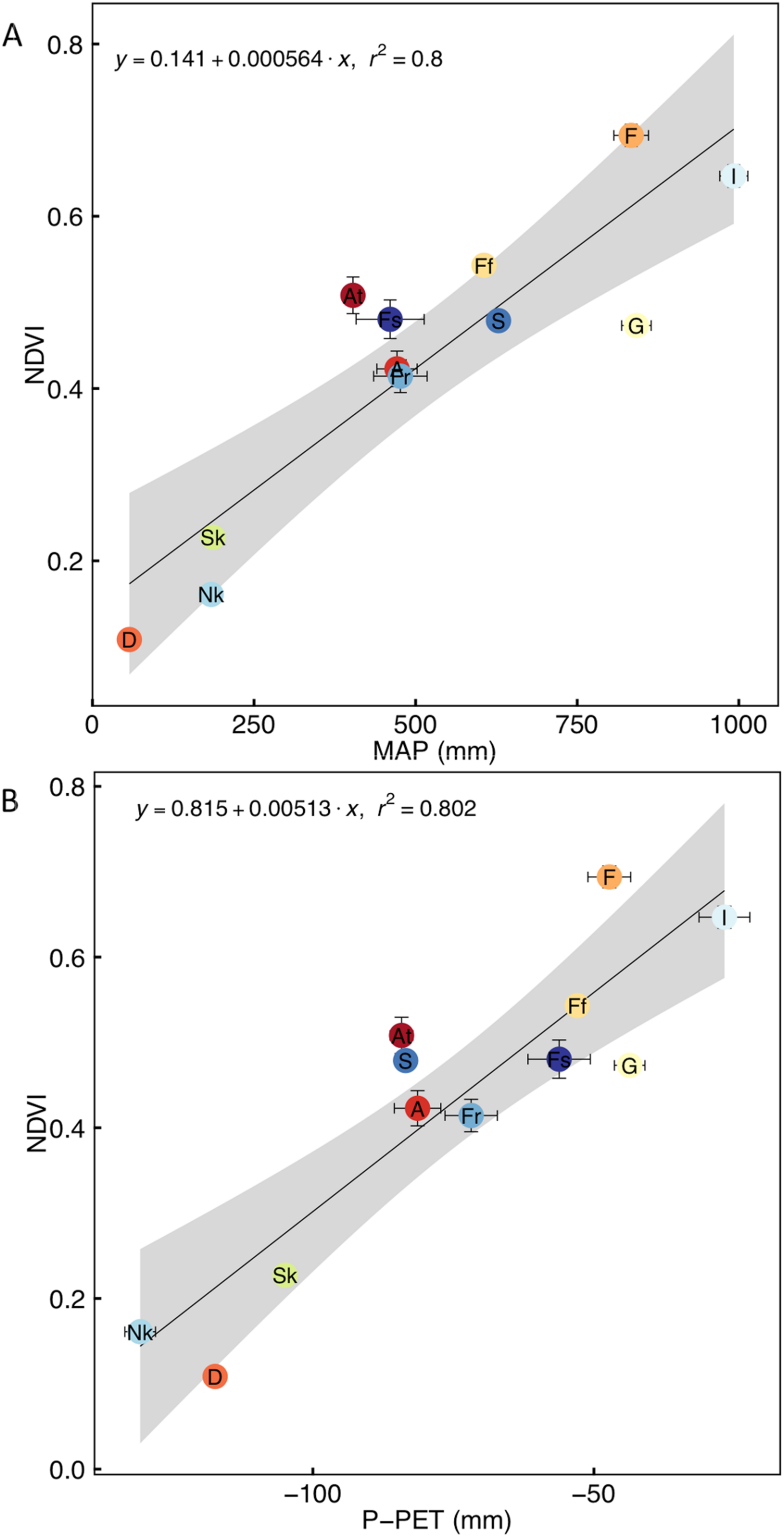
Correlation of annual average normalised difference vegetation index (NDVI) with (A) the average annual precipitation (MAP) and (B) water availability measured as the average of monthly precipitation (mm)–potential evapotranspiration (PET, mm) of each vegetation type. The vegetation types are indicated by codes (see Table 1). The coefficient of determination (r^2^) is shown for significant relationships (P < 0.05). Points and error bars indicate mean ± SE (for n see Table 1) and the grey bands indicate the 95% confidence limits.

NDVI values had high intra-annual variability in Savanna and Grassland, but much less so for other vegetation, with fynbos being the least variable (S1 Fig). Although MAT is not linearly related to the average annual NDVI of vegetation types (r^2^ < 0.01; data not shown), MAP is a strong linear predictor of this (Fig 2), with Forest and Indian Ocean Coastal Belt vegetation having high NDVI values that are distinct from those of the more mesic vegetation types (Grassland, Albany Thicket, Azonal, fynbos, renosterveld, strandveld), which are in turn distinct from the more arid vegetation types (Succulent Karoo, Nama-Karoo and Desert). Water availability, measured as precipitation-PET (P–PET; S2 Fig), is an equally strong predictor of averaged annual NDVI (Fig 2). Water availability is significantly positively related to MAP (r^2^ = 0.84, data not shown) and negatively to PET (r^2^ = 0.50; data not shown). Grasslands have an anomalously low NDVI and Albany Thicket and strandveld a high NDVI for the amount of MAP. Grassland also had low NDVI relative to water availability while Forest, Albany Thicket and Savanna had a high NDVI for the water availability. This indicates that something other than MAP or water availability influences the NDVI of these vegetation types (e.g. edaphic properties or disturbances such as fire or herbivory; see below). PCI was not significantly related to NDVI (r^2^ = 0.10; data not shown).

**Fig 3:**
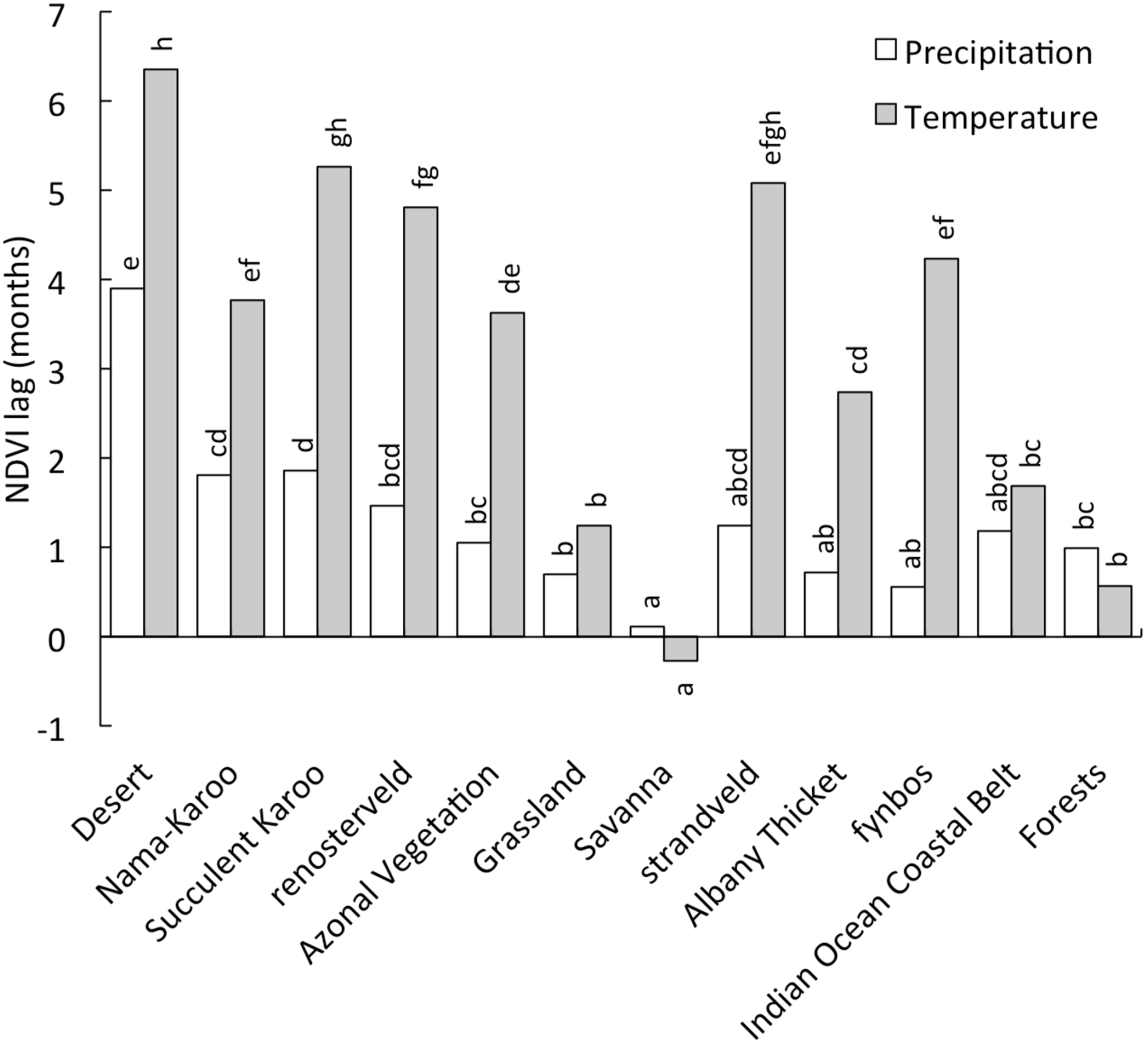
Time lag between maximum monthly precipitation or mean monthly temperature and maximum normalised difference vegetation index (NDVI) for each vegetation type. Different letters indicate significant (P < 0.05) differences between vegetation types as determined by one-way ANOVA followed by post-hoc Tukey tests. Vegetation types are arranged in order of increasing NDVI.

The co-occurrence of annual precipitation and temperature maxima with NDVI maxima was measured by estimating the lag (months, S1 Table) between these maxima (Fig 3). Savanna NDVI lags maximum precipitation only slightly, whereas Desert has relatively long periods between maximum precipitation and NDVI. NDVI lags temperature to a similar extent in fynbos, strandveld and renosterveld, whereas Succulent Karoo had significantly longer lag periods than fynbos. Overall, the lags between NDVI and precipitation are small, and shorter than those between NDVI and temperature, indicating that in this temperate region, precipitation is likely more limiting than temperature.

### Edaphic predictors of NDVI

**Fig 4:**
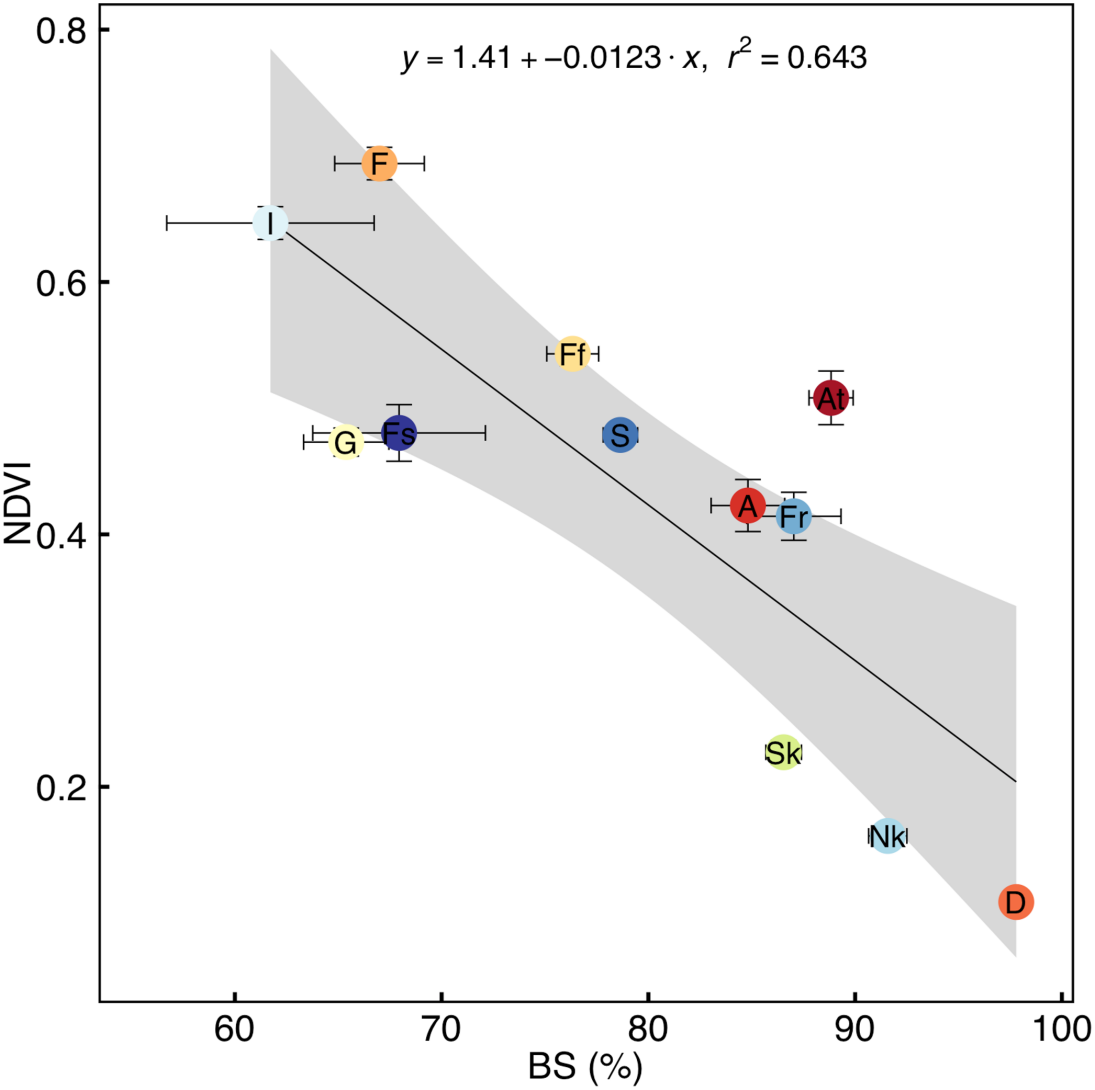
Correlation of annual average normalised difference vegetation index (NDVI) with base saturation (BS) of each vegetation type. The vegetation types are indicated by codes (see Table 1). The coefficient of determination (r^2^) is shown for significant relationships (P < 0.05). Points and error bars indicate mean ± SE (for n see Table 1) and the grey band indicates the 95% confidence limits.

**Fig 5:**
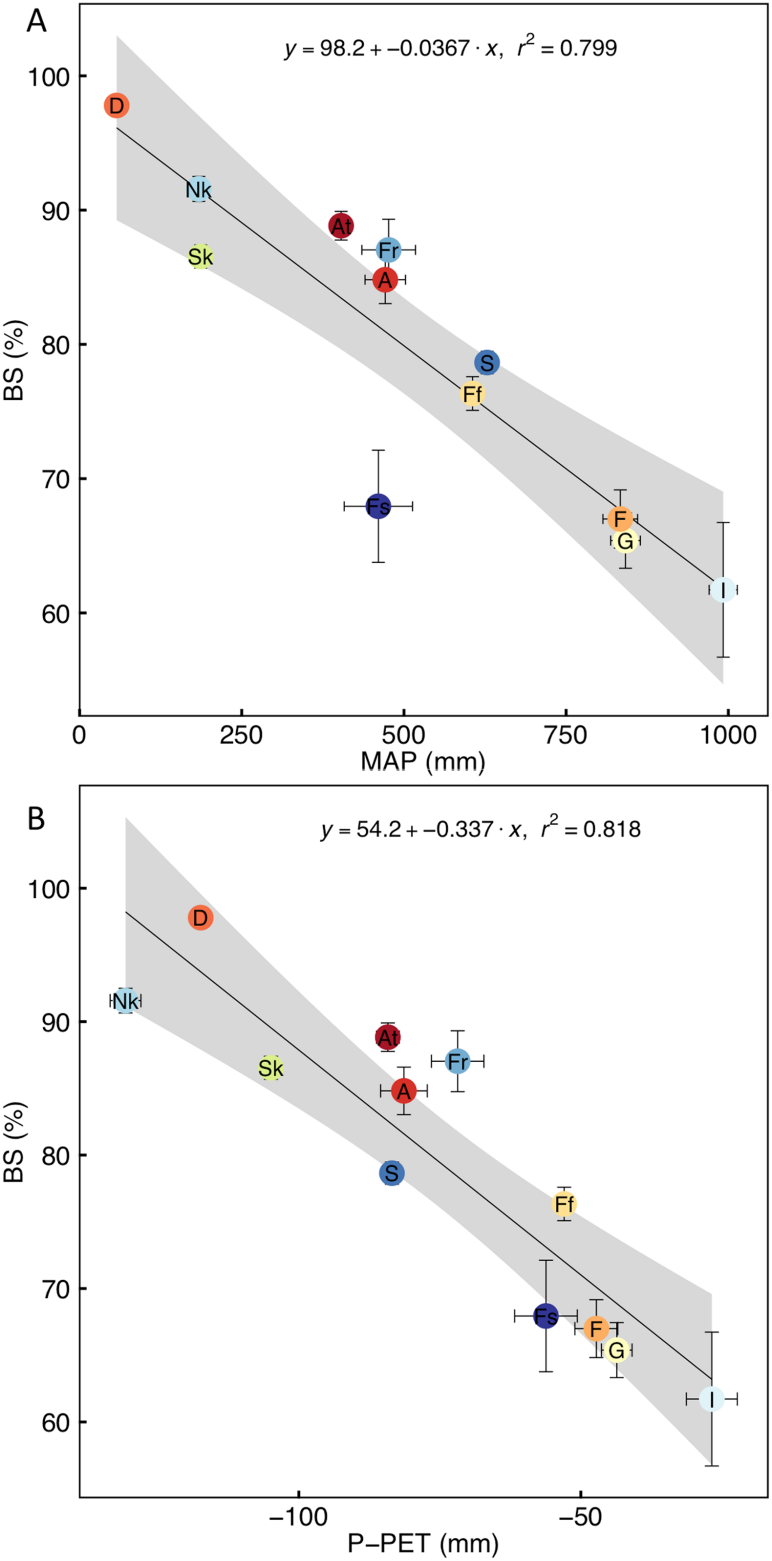
Correlation between base saturation (BS) and (A) the average annual precipitation (MAP) and (B) water availability measured as the average of monthly precipitation—potential evapotranspiration (PET) of each vegetation type (Fynbos separated into strandveld, renosterveld and fynbos). The vegetation types are indicated by codes (see Table 1). The coefficient of determination (r^2^) is shown for significant relationships (P < 0.05). Points and error bars indicate mean ± SE (for n see Table 1) and the grey bands indicate the 95% confidence limits.

**Fig 6:**
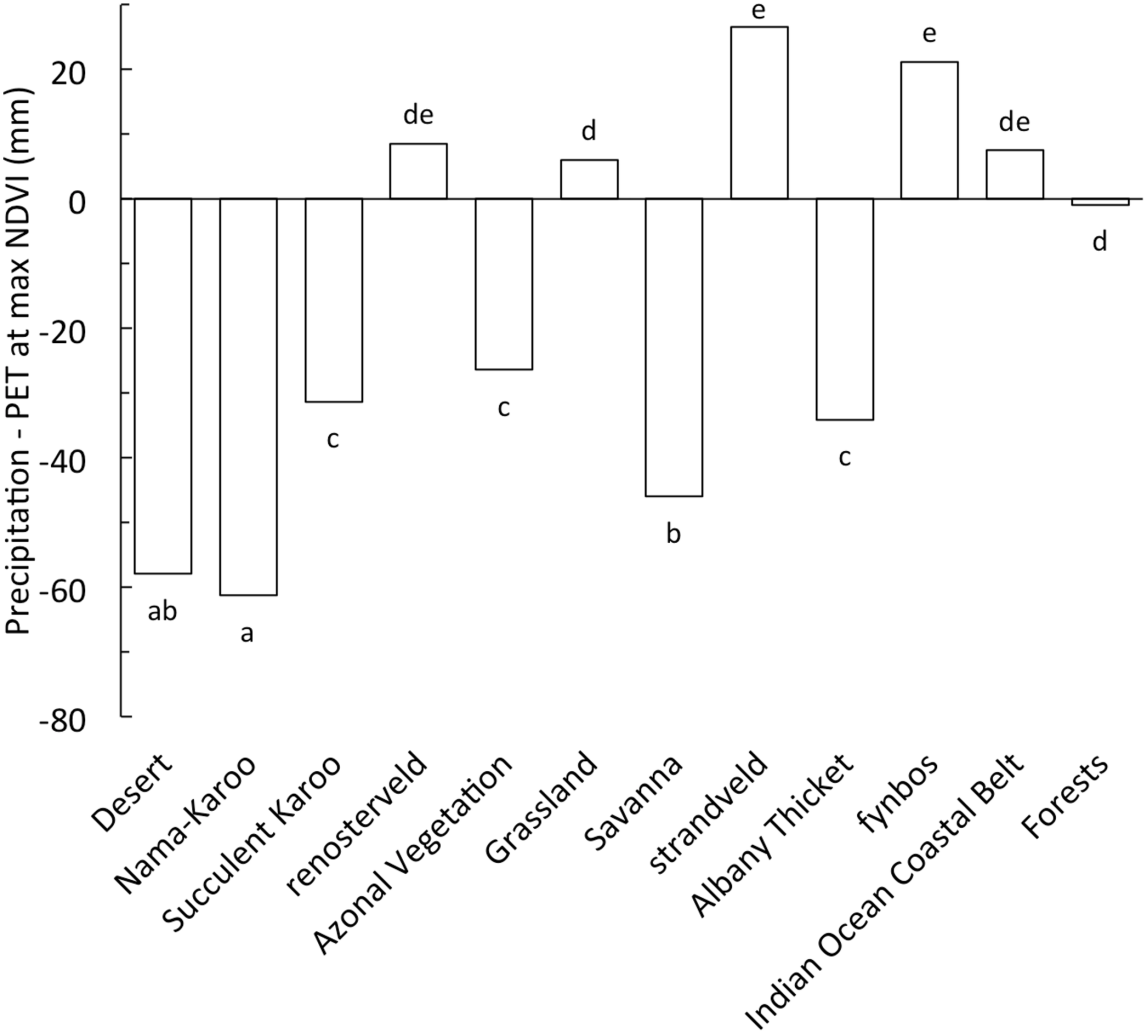
Water availability measured as the average of monthly precipitation—potential evapotranspiration (P-PET) for the vegetation types in the month when annual NDVI was maximal. A positive value indicates that precipitation exceeded potential evaporation when NDVI was maximal. Different letters indicate significant (P < 0.05) differences between vegetation types as determined by one-way ANOVA followed by post-hoc Tukey tests. Vegetation types are arranged in order of increasing NDVI.

Base saturation is a negative linear predictor of average annual NDVI of the vegetation types (Fig 4). Albany Thicket has an anomalously high NDVI for BS and for water availability (Fig 2), indicating that some extraneous environmental factors (e.g. lack of fire, see data in Archibald et al. [59]) possibly drives greater NDVI of this vegetation. A similar set of relationships exists for pH with average annual NDVI for the vegetation types (data not shown), but not for other edaphic variables. More mesic vegetation including Indian Ocean Coastal Belt, Grassland, Forest and strandveld have low BS and high NDVI. In contrast arid Succulent Karoo, Nama-Karoo and Desert have high BS and low NDVI. Base saturation is negatively related to both MAP (Fig 5A) and water availability (Fig 5B) indicating that water availability increases soil leaching. In contrast to the relationship between BS and P–PET, TEB was unimodal with P–PET, increasing up to a maximum when water availability was *ca*. -65 mm per annum, and then decreasing (S3 Fig). Annual precipitation exceeds PET for fynbos, renosterveld, strandveld, Grassland and Indian Ocean Coastal Belt at a time when NDVI is maximal (Fig 6), indicating an excess of water potentially at a time of maximum plant demand for water, possibly contributing to soil leaching. In contrast, Savanna, Nama-Karoo and Desert have water deficits during periods of high NDVI.

### Evidence for interactions

Multiple regression analysis following exclusion of collinear predictors retained climatic (i.e. P-PET, precipitation seasonality and PCI) and edaphic variables (i.e. BS, TEB, pH, CEC and OC) as predictors of the overall NDVI across all vegetation types (Table 2). Climatic predictors of BS that were retained following model simplification included P-PET, precipitation seasonality and PCI, TEB, CEC and OC. When each vegetation type was analysed separately, the standardised coefficients for multiple regression analysis indicated that both P-PET and BS were correlated with NDVI for most vegetation types (Table 3). The only vegetation for which BS did not enter the model was Nama-Karoo, indicating that within this vegetation P-PET is a more important constraint on NDVI than BS.

**Table 2:** Multiple regression analysis of the climatic and edaphic predictors of NDVI and BS retained following stepwise model simplification based on AIC.

	NDVI		BS	
Variable	r^2^/Coefficient	P	r^2^/Coefficient	P
**Model**	**0.69**	**<0.001**	**0.90**	**<0.001**
**Intercept**	**-0.02**	**0.746**	**0.10**	**<0.001**
P-PET	0.71	<0.001	-0.18	<0.001
P-PET^2^	-0.15	<0.001	0.05	<0.001
PCI	0.21	<0.001	-0.04	<0.001
P seasonality	-0.31	<0.001	-0.02	<0.001
P seasonality^2^	0.12	<0.001		
BS	-0.50	<0.001		
OC	0.60	<0.001	0.16	<0.001
OC^2^	-0.04	<0.001		
pH	0.23	<0.001		
pH^2^	-0.11	<0.001		
TEB	1.45	<0.001	3.33	<0.001
TEB^2^			-0.25	<0.001
CEC	-1.49	<0.001	-2.86	<0.001

The standardized coefficients for each variable are shown with their P for inclusion in the model. The r^2^ and P for the overall models and intercepts are also included (bold).

**Table 3:** Comparison of the multiple regression standardized coefficients for average annual NDVI regressed on water availability (P-PET) and base saturation (BS) for the vegetation types of South Africa, as defined by Mucina and Rutherford (2006), but with the Fynbos separated into strandveld, renosterveld and fynbos.

Vegetation	P-PET	(P-PET)^2^	BS	BS^2^	r^2^	P	n
Savanna (S)	-0.52	0.84	-0.80	0.32	0.67	<0.001	339
Nama-Karoo (Nk)	0.84				0.69	<0.001	47
Grassland (G)	-1.08	-1.44	1.18	-0.50	0.61	<0.001	119
Forests (F)		0.23		-0.73	0.41	<0.001	84
Indian Ocean Coastal Belt (I)						n.s.	16
Albany Thicket (At)	-2.71	-0.29		-1.97	0.67	<0.001	65
Azonal (A)	-1.22	1.56	-1.84	-0.75	0.53	<0.001	79
Desert (D)	0.35			0.56	0.21	0.015	31
fynbos (Ff)		-0.30	-0.45		0.39	<0.001	206
renosterveld (Fr)		-0.68			0.45	<0.001	32
Succulent Karoo (Sk)	-4.94		4.67	0.72	0.67	<0.001	86
strandveld (Fs)		-8.86	8.99	0.73	0.83	<0.001	12

The values are arranged from predominantly summer to predominantly winter-precipitation. Included are the overall model r^2^ and P values (n.s. = not significant) with number of replicates per vegetation type (n).

**Fig 7:**
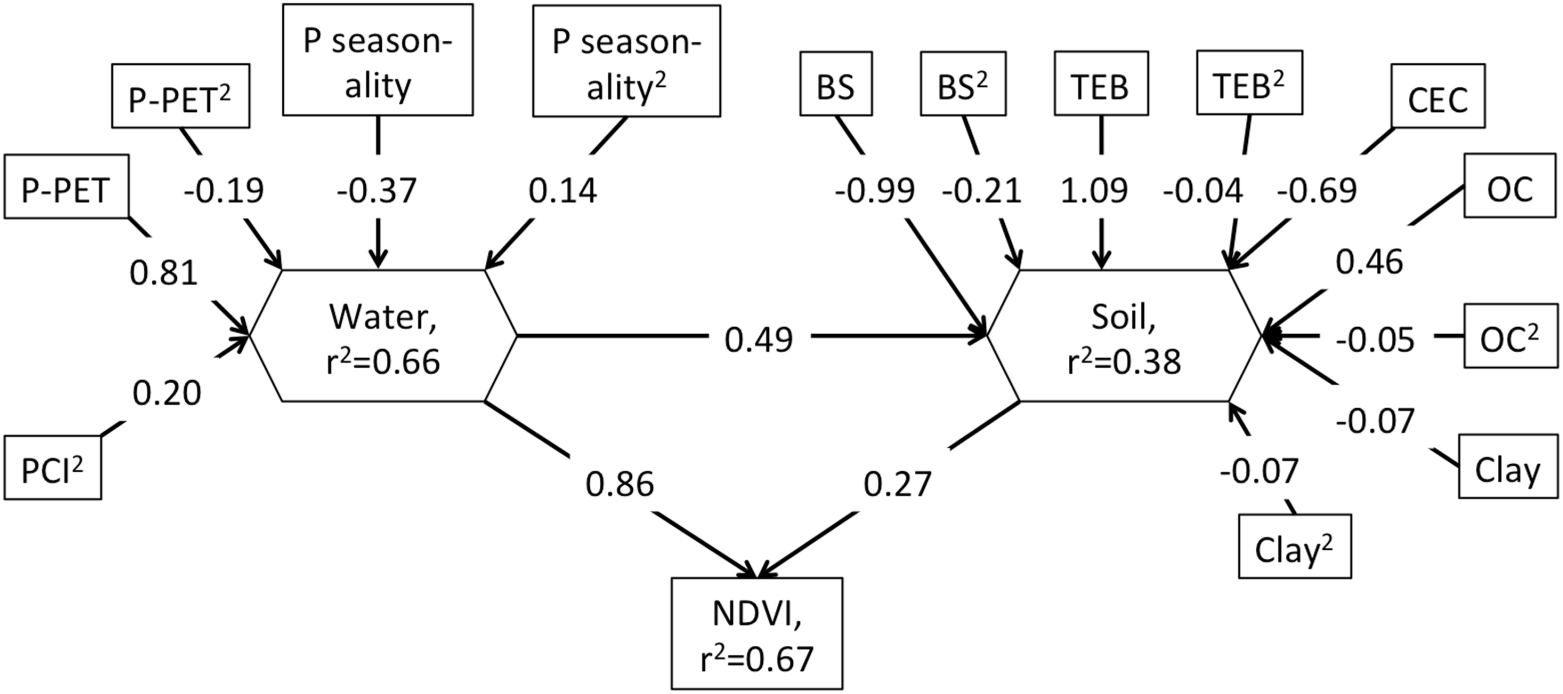
SEM representing the standardised path coefficients and r^2^ values for significant (P < 0.001) paths from exogenous environmental variables including water availability measured as the average of monthly precipitation—potential evapotranspiration (P-PET), precipitation seasonality, precipitation concentration index (PCI), base saturation (BS), total exchangeable bases (TEB), cation exchange capacity (CEC), organic carbon (OC) and clay through two composite variables (Water and Soil) to NDVI. The direct influence of water availability on NDVI (0.86) is greater than the indirect influence through the soil composite variable (product of coefficients = 0.13). The Chi^2^ P value was 0.97 with d.f = 1.

The simplified BRT model for NDVI included 5 000 trees with 87.5% of deviance explained. The relative percentage contributions of predictor variables in the simplified model for NDVI were P–PET (51%), PCI (14%), precipitation seasonality (12%), MAT (10%), BS (4%), pH (3%), TEB (3%) and clay (3%). The simplified BRT model for BS included 5 000 trees with 97.8% of deviance explained. The relative percentage contributions of the predictor variables in the simplified model for BS were TEB (50%), OC (20%), P–PET (17%), CEC (8%), clay (3%), precipitation seasonality (1%) and MAT (1%), and PCI (1%). When soil variables were excluded from the BRT model of BS (3 900 trees explaining 84.1% of deviance) the percentage contributions of the predictor variables were P-PET (44%), precipitation seasonality (22%), PCI (18%) and MAT (16%), indicating that although precipitation seasonality is secondary to overall water availability, it does play a significant role in leaching. The SEM verified that the direct effects of a composite of indicators of water availability had a stronger association with NDVI than did water availability operating through a composite of soil variables (Fig 7). While the effect of the soil composite was 31% of the effect of the water composite on NDVI, the indirect effect of water through the soil composite was 16% of the direct effect of water on NDVI.

**Fig 8:**
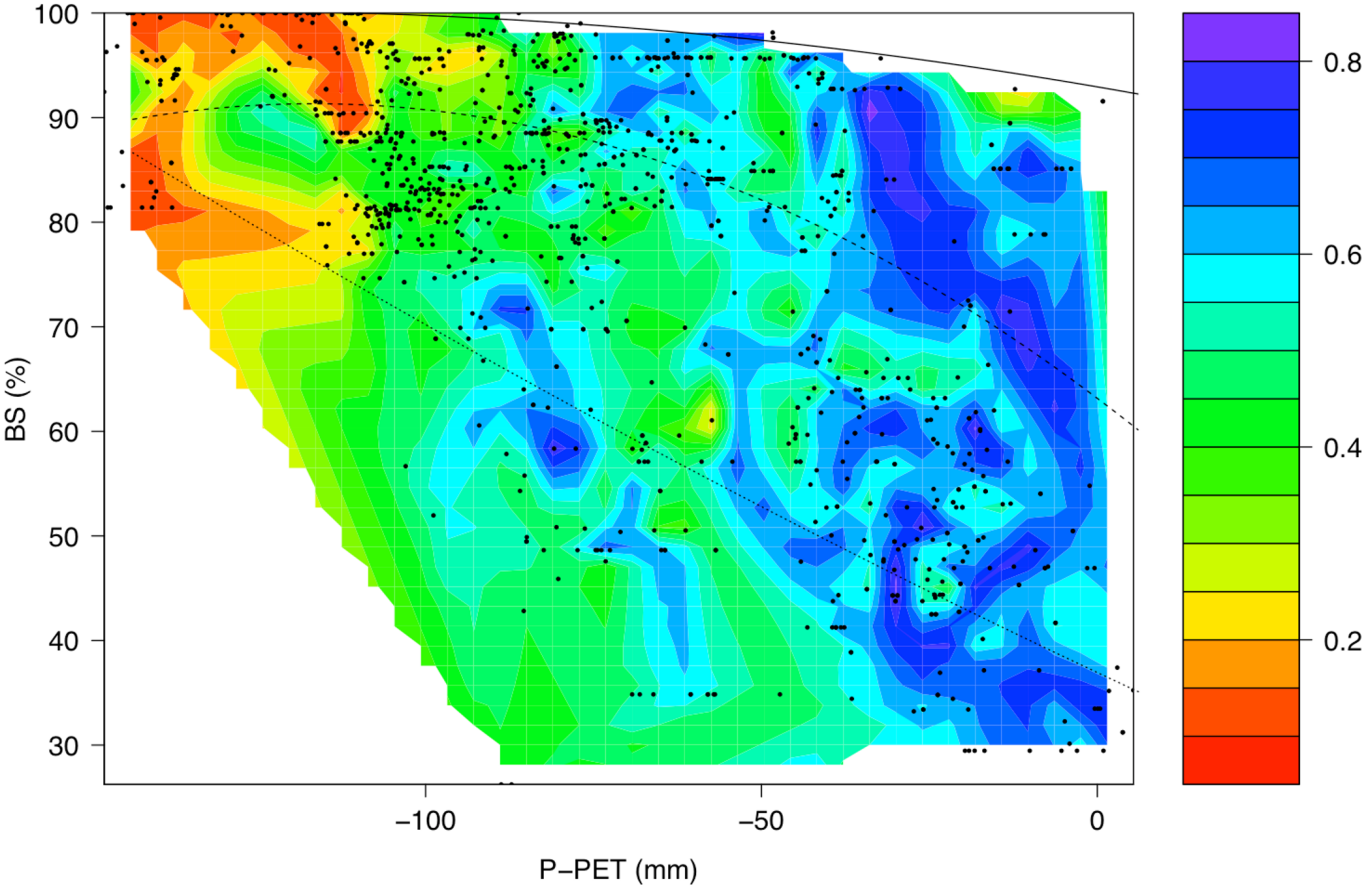
Contour plot of the average annual NDVI versus water availability (P-PET) and base saturation (BS) with a superimposed scatterplot for the entire dataset. The colours of the contour surface correspond to the NDVI values of the points (key on right). The solid, dashed and dotted lines represent the 95%, 50% and 5% quadratic quantiles, respectively, estimated using the ‘quantreg’ package [60] in R [57].

There are no sites in this analysis with the combination of both low P-PET and low BS, and few with high P-PET and high BS, suggesting that these conditions are mutually exclusive (Fig 8). High NDVI’s are most common with both high P-PET and low BS, although some sites with high NDVI occur at moderate P-PET and BS (e.g. -80 mm P-PET and 58% BS). This may imply that for some sites high P-PET (i.e. *ca*. ≥ -25 mm) may partially relieve the limitations of low BS, and that higher BS may also partially reduce the dependence of NDVI on P-PET. The scatter of points, however, also illustrates that other factors omitted from this analysis (cf. Table 2) and variables not considered in this study (e.g. fire and disturbance) may also contribute to determining NDVI.

## Discussion

Extended periods of time without glaciation have been associated with the development of highly leached soils in mediterranean-ecosystems (i.e. OCBILs; [31]), but leaching is the consequence of sustained runoff and percolation of water through soils [24]. The influence of winter-precipitation on BS in multiple regression, BRT and structural equation models indicates that it is not only the volume of precipitation that is relevant, but also the seasonality and concentration (i.e. PCI) of that precipitation that governs soil water fluxes. Regionally, it is more common in winter- than in summer-precipitation areas for water to exceed evapotranspirational demand, contributing to surface runoff and percolation into vadose and groundwater zones resulting in leaching. Base saturation is a strong predictor of soil fertility for crops and forests [61] and provides a measure of leaching that is relatively independent of soil cation exchange capacity or clay content, since it measures the proportion of exchange sites in the soil that are occupied by the basic cations (i.e. Ca, Mg, K and Na). As a consequence, BS is generally highly correlated with pH [62], as is also the case here (r^2^ = 0.83, data not shown). High water availability thus both promotes soil development (e.g. accumulation of soil C and N) and results in low BS and pH. As a consequence, soils derived from distinct geologies may have similar BS values at similar MAP (e.g. Savanna and fynbos). Thus, although geological origins and time are important determinants of the degree of leaching of soils, the intensity of leaching, as determined by seasonal water availability, must also be considered.

Soil fertility for plant growth is the outcome of many interacting pedogenic factors [22], apart from leaching. For example, vegetation biomass follows a unimodal curve along soil chronosequences [63] due to changes in soil nutrients that include the accumulation of some nutrients (e.g. N) and the depletion of others (e.g. cations and P; [64, 65, 27]). The intensity with which these pedological factors operate is dependent on climate, with greater water availability increasing the rate at which these processes occur. Albrecht’s curve [23] leads to the expectation of increased BS with increasing water balance to the point where MAP = PET, and decreasing BS where MAP > PET [24]. In contrast to this expectation, BS in this study declines consistently with increasing water availability. However, the variation in TEB with P-PET follows a unimodal curve, as also reported for Africa by Huston [24], except that the maximum TEB defined by the 95% quantile line occurs below P–PET = 0. The large scatter of points below the quantile line indicates that P-PET only limits the maximum TEB, whereas the actual TEB is also determined by other factors (e.g. differing geologies, ages, developmental history; [24]). While it might be conceptually appealing to expect the maximum TEB at P–PET = 0, this is a rather arbitrary expectation based on studies undertaken in North America [23] that neglect the duration and historical climate under which pedogenesis occurred. For example, the reason that TEB is maximum at close to P–PET = 0 in North America and in Europe, but occurs at P–PET<0 for Africa and Australia is probably related to the fact that the latter two continents both have rather low values of P–PET [24] and have been relatively undisturbed by glaciation, providing time for slow leaching effects to accrue over long periods of time. The decrease in soil nutrient availability with water availability is likely associated with a suite of plant traits that enable nutrient acquisition and resource-use efficiency, and explains why these traits may be co-correlated with precipitation reliability, that is in turn related to winter-precipitation [5].

The expectation that vegetation biomass increases monotonically with MAP [66, 67, 68, 69, 70] has been stated as a first-order “capacity” rule in which the geographic variation in water-energy dynamics is matched by the geographic variation in the intensity and duration of photosynthesis [71]. This has been questioned by Schuur [72] who found a negative association between MAP and NPP in humid ecosystems. Although NPP is not directly equivalent to NDVI, it is generally calculated from NDVI with other measures [73]. We present evidence that the increases in NDVI driven by water availability occur despite water availability-driven decreases in BS, which has the consequence that increases in NDVI at high water-availability may be limited by declining BS. Despite this putative limitation imposed by declining BS on NDVI, the correlation of NDVI with MAP remains strong, both regionally and for many individual vegetation types. We suggest that this is because higher availability of water improves access to nutrients [37, 39], and conversely, high nutrient availability reduces the requirement for water. This trade-off between water and nutrient availability may enable NDVI to increase with MAP, despite decreases in available nutrients, resulting in BS being a weaker predictor of NDVI.

The strong link between water availability and NDVI globally [41] is particularly important in South Africa, where temperatures are relatively moderate and not as strongly associated with NDVI as water availability (Fig 3). The seasonality of precipitation does not strongly influence average annual NDVI, as evidenced by the component vegetation of the predominantly winter-precipitation vegetation types (fynbos, renosterveld, strandveld and Succulent Karoo) all being close to the regional (i.e. South African) regression lines for NDVI with MAP and P-PET. For example, the largely summer-precipitation Nama-Karoo region, which has similar aridity to the largely winter-precipitation Succulent Karoo, also has similar NDVI values. Likewise, largely winter-precipitation fynbos receives similar MAP to summer-precipitation Savanna and only slightly higher average NDVI values, despite intra-annual NDVI-variability being much lower in fynbos than in Savanna. The predominantly winter-precipitation fynbos and Succulent Karoo do, however, have higher water availability (i.e. P–PET) than the Savanna and Nama-Karoo, respectively. Thus, despite similar MAP, winter-precipitation results in greater water availability than does summer-precipitation. The fact that winter-precipitation region NDVIs are similar to those of the comparable (based on MAP) summer-precipitation regions, however, indicates that plants are not able to capitalise on this greater water availability by producing leafier biomass. In contrast, evaporation of water in summer is likely to reduce water availability for plant transpiration. The lack of strong differences in NDVI between summer- and winter-precipitation areas with similar amounts of precipitation indicates that these two divergent conditions offer distinct ecophysiological challenges for the floras, that are met with almost equal success. This does not mean that summer-droughts are not a constraint on leafy biomass in the GCFR [17] but that summer-rains also have attendant limitations.

In summer-precipitation areas water, temperature and light conditions appropriate for growth are loosely coincident, whereas resource availabilities and growth may be partially asynchronous for winter-precipitation-ecosystem vegetation [4]. Within the predominantly winter-precipitation regions, the lag between NDVI and precipitation maxima was, however, particularly short for fynbos (0.6 months), indicating that this vegetation utilizes the higher water and nutrient availability in winter to green-up. Greening is not necessarily tightly associated with growth [74]. For example, deep-rooted components of the winter-precipitation-ecosystem flora (16, 17) and succulents (Succulent Karoo) that store water and nutrients [75] do continue to grow in spring and summer. That plants capitalise on wet winter periods is consistent with the suggestion that small leaves in this vegetation have the dual role of ensuring transpirational water loss and nutrient transport in winter, but also allowing plants to tolerate summer droughts by improving heat dissipation [14]. Thus at least some components of fynbos are able to utilise winter-precipitation for greening in winter, and have specific adaptations that allow this, forcing a degree of trait-convergence in the global winter-precipitation ecosystem vegetation.

## Conclusions

The coincidence of highly leached soils with winter-precipitation-ecosystems is partially because winter-precipitation is particularly leaching, relative to summer-precipitation, providing a powerful link between nutrient availability and climate. The high leaching potential of the winter-precipitation climate is thus likely to contribute to edaphic diversity, by providing a high-intensity transformative component of pedogenesis, superimposed on variable geologies and soil ages. The negative consequences of leaching for plant growth are, however, partially mitigated by trade-offs that may exist between water availability and nutrients, and by diverse adaptations to nutrient limitation. We thus suggest that in addition to prolonged periods of leaching (i.e. OCBIL; [31]), winter-precipitation is a contributor to nutrient impoverishment of soils and that it is no coincidence that two of the most infertile landscapes in the world that exhibit growth-form convergence (i.e. south western South Africa and Australia) are both winter-precipitation ecosystems, that should perhaps rather be referred to as old mediterranean infertile landscapes (OMILs).

## Supporting Information

S1 Fig The intra-annual variability of NDVI (variability = (max-min)/min) for each vegetation type (Fynbos separated into strandveld, renosterveld and fynbos). Different letters indicate significant (P < 0.05) differences between vegetation types as determined by one-way ANOVA followed by post-hoc Tukey tests.
(TIF)

S2 Fig Map of the variation in annual averages of monthly water availability (precipitation—potential evapotranspiration, P-PET, mm) for South Africa. National and provincial borders are indicated.
(TIF)

S3 Fig Variation of total exchangeable bases (TEB) with water availability (P-PET) for all the individual sites sampled. The solid, dashed and dotted lines represent the 95%, 50% and 5% quadratic quantiles, respectively, estimated using the ‘quantreg’ package [60] in R [57].
(TIF)

S1 Table The average ± SE for each South African vegetation type of the months during which temperature (T), precipitation (P), normalized difference vegetation index (NDVI) and precipitation—potential evapotranspiration (P–PET) were maximum. Vegetation types are as defined by Mucina and Rutherford [8], but with the Fynbos separated into strandveld, renosterveld and fynbos. Different letters indicate significant (P < 0.05) differences between vegetation types as determined by one-way ANOVA followed by post-hoc Tukey tests. The values are arranged from predominantly summer to predominantly winter rainfall (Table 1).
(DOCX)
