# Supplementary figures and images for: The Consequences of Precipitation Seasonality for Mediterranean-Ecosystem Vegetation of South Africa
Source: PLoS One. 2015 Dec 9;10(12):e0144512. doi: 10.1371/journal.pone.0144512 (PMC4674101; doi:10.1371/journal.pone.0144512)

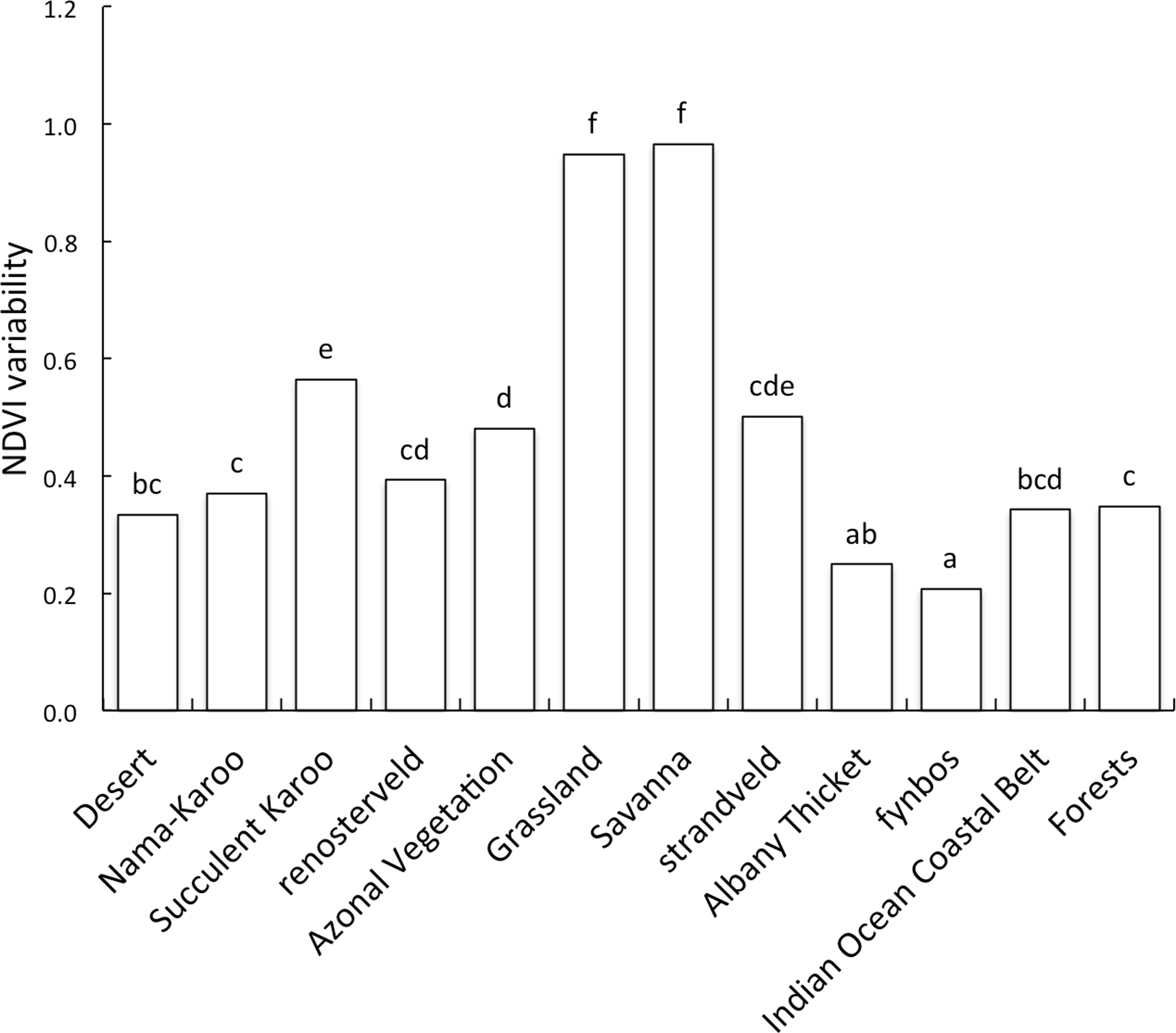

Supplement: S1 Fig — Different letters indicate significant (P < 0.05) differences between vegetation types as determined by one-way ANOVA followed by post-hoc Tukey tests. (TIF) [file pone.0144512.s001.tif]

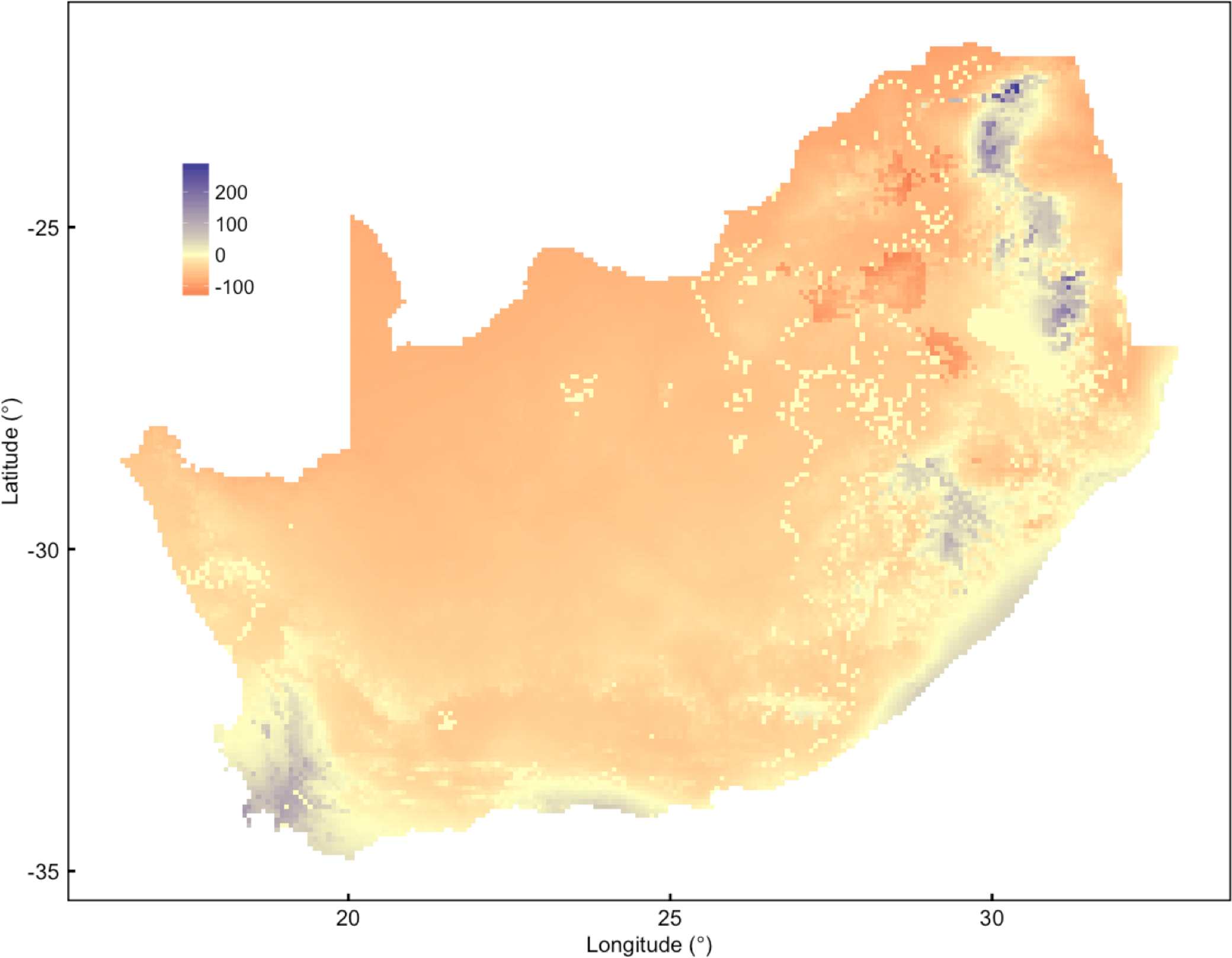

Supplement: S2 Fig — National and provincial borders are indicated. (TIF) [file pone.0144512.s002.tif]

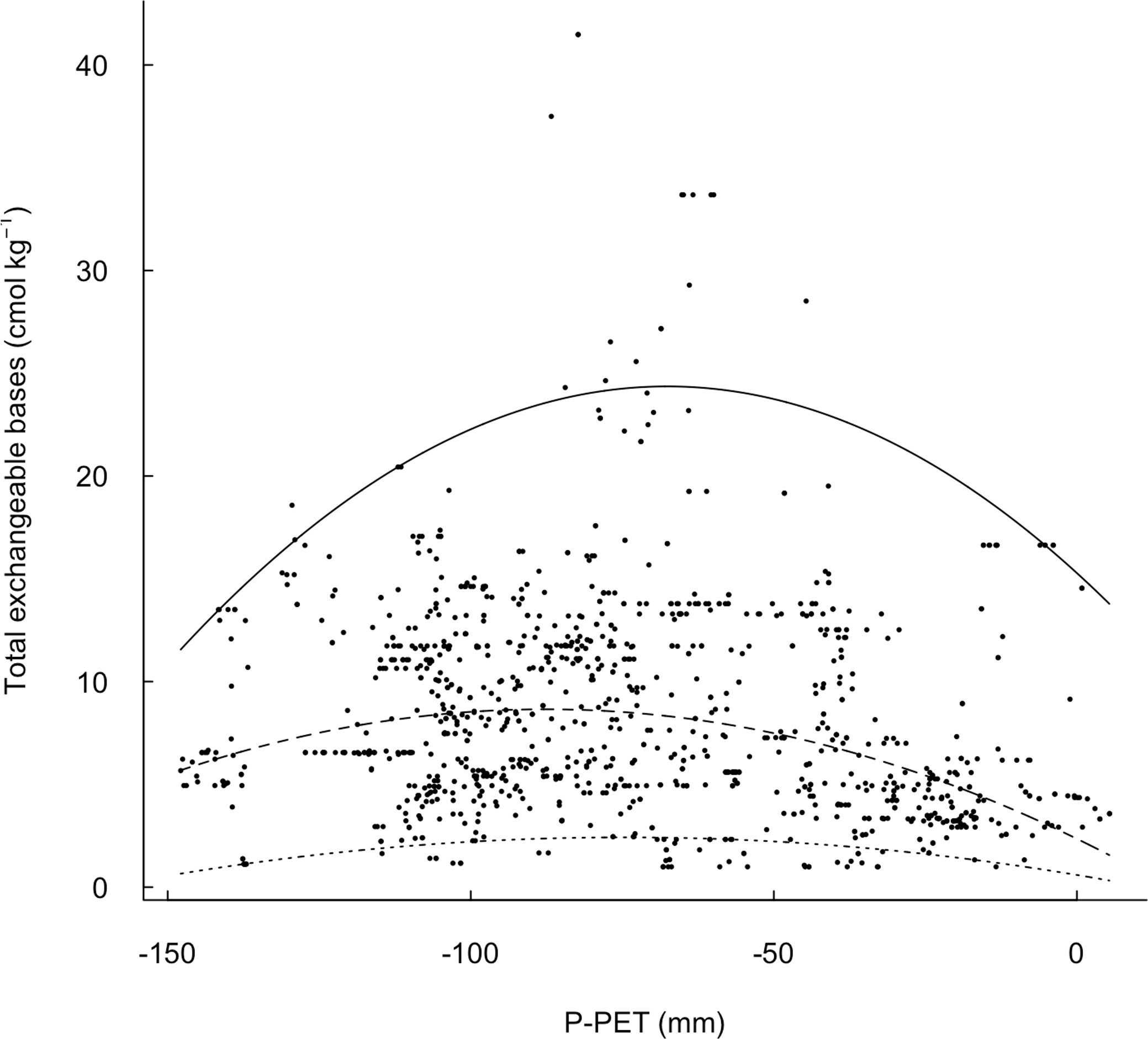

Supplement: S3 Fig — The solid, dashed and dotted lines represent the 95%, 50% and 5% quadratic quantiles, respectively, estimated using the ‘quantreg’ package [60] in R [57]. (TIF) [file pone.0144512.s003.tif]
