# Supplementary material for: The Consequences of Precipitation Seasonality for Mediterranean-Ecosystem Vegetation of South Africa
Source: PLoS One. 2015 Dec 9;10(12):e0144512. doi: 10.1371/journal.pone.0144512 (PMC4674101; doi:10.1371/journal.pone.0144512)
Supplement: S1 Table — Vegetation types are as defined by Mucina and Rutherford [8], but with the Fynbos separated into strandveld, renosterveld and fynbos. Different letters indicate significant (P < 0.05) differences between vegetation types as determined by one-way ANOVA followed by post-hoc Tukey tests. The values are arranged from predominantly summer to predominantly winter rainfall (Table 1). (DOCX) [file pone.0144512.s004.docx]

| **Vegetation** | **Max T** | **Max P** | **Max NDVI** | **Max P–PET** |
| --- | --- | --- | --- | --- |
| Savanna Biome | 2.87 ± 0.22 c | 2.49 ± 0.18 b | 2.6 ± 0.04 ab | 2.78 ± 0.10 a |
| Nama-Karoo Biome | 1.04 ± 0.03 a | 3.00 ± 0.01 bcd | 4.81 ± 0.1 de | 6.00 ± 0.01 d |
| Grassland Biome | 1.18 ± 0.10 a | 1.72 ± 0.21 a | 2.42 ± 0.07 a | 3.34 ± 0.22 b |
| Forests | 3.07 ± 0.42 c | 2.65 ± 0.31 bc | 3.64 ± 0.17 c | 3.2 ± 0.22 b |
| Indian Ocean Coastal Belt | 1.38 ± 0.13 ab | 1.88 ± 0.20 ab | 3.06 ± 0.23 b | 2.38 ± 0.13 a |
| Albany Thicket Biome | 1.85 ± 0.05 ab | 3.86 ± 0.29 de | 4.58 ± 0.11 d | 5.97 ± 0.02 d |
| Azonal Vegetation | 1.35 ± 0.05 ab | 3.94 ± 0.29 e | 4.99 ± 0.24 e | 4.89 ± 0.20 c |
| Desert Biome | 1.10 ± 0.05 ab | 3.55 ± 0.21 cde | 7.45 ± 0.1 h | 6.00 ± 0.01 d |
| fynbos | 1.90 ± 0.02 b | 5.58 ± 0.15 f | 6.14 ± 0.08 g | 6.06 ± 0.04 d |
| renosterveld | 1.78 ± 0.07 ab | 5.13 ± 0.32 f | 6.59 ± 0.18 f | 6.03 ± 0.03 d |
| Succulent Karoo Biome | 1.83 ± 0.04 ab | 5.23 ± 0.14 f | 7.09 ± 0.08 h | 6.01 ± 0.01 d |
| strandveld | 2.00 ± 0.01 abc | 5.83 ± 0.17 f | 7.08 ± 0.15 fh | 6.00 ± 0.01 d |
